# Features of entropy optimization on MHD couple stress nanofluid slip flow with melting heat transfer and nonlinear thermal radiation

**DOI:** 10.1038/s41598-020-76133-y

**Published:** 2020-11-05

**Authors:** F. Mabood, T. A. Yusuf, Gabriella Bognár

**Affiliations:** 1 Department of Information Technology, Fanshawe College London, London, ON N5Y 5R6 Canada; 2 Department of Mathematics, University of Ilorin, Ilorin, Kwara State Nigeria; 3 University of Miskolc, Miskolc-Egyetemváros, 3515 Hungary

**Keywords:** Engineering, Mechanical engineering

## Abstract

Numerical analysis is performed for magnetohydrodynamics (MHD) couple stress nanofluid flow over a stretching sheet with melting and nonlinear radiation. The second law of thermodynamics is also incorporated with first-order slip. Nanofluid characteristics for thermophoresis and Brownian moments are encountered. The system that comprises differential equations of partial derivatives is remodeled into the system of differential equations via similarity transformations and then solved numerically through the Runge–Kutta–Fehlberg fourth-fifth (RKF-45) order technique. The physical parameters, which emerges from the derived system are discussed in graphical format. The significant outcomes of the current investigation are that the velocity field decays for a higher magnetic parameter. Another, important outcome of the study is both temperature and concentration are increasing functions of the first-order slip. Nusselt and Sherwood numbers are decreasing with an increase in magnetic strength. Further, Bejan number augment due to enhancement in the first-order slip and couple stress fluid parameters whereas a differing tendency is shown for magnetic and radiation parameters.

## Introduction

By the idealization of continuum mechanics, the theory of Couple stress fluid is based on a classical theory of viscous Newtonian fluids such fluids include long-chain additives lubricant oil, polymeric suspensions, blood, rheological complex fluids and many more. An earlier study by Stokes’^1^ has reported an in-depth survey of general theories and rheological behavior of couple stresses fluids. From then, more investigations concerning the flow of couple stress fluid over different geometries and under various flow parameters can be found in Refs.^2–8^.

Several authors have shown their keen interest in heat transfer phenomena and fluid flow. The mechanism of transportation of heat during the melting process is quite phenomenal for the acceptable quality product in industrial and engineering processes. This plays a significant role in the industries such as welding and magma solidification, casting, permafrost melting and thawing of frozen ground, etc. In light of these, Hayat et al.^9^ deal with the stagnation points of couple stress fluid with melting heating. The computational fluid dynamics modelling of three types of nanofluid flows are studied for the Blasius problem in the paper^10^. The heat and mass transfer on MHD flow over a porous stretched surface using HAM has been studied by Jitender et al.^11^. Similarity solution for boundary layer MHD flow of non-Newtonian fluids over a nonlinearly stretching flat surface was reported by Bognár and Hriczó^12^. Mabood and Mastroberardino^13^ extended the study involving the boundary layer fluid with nanomaterials over a warm stretching surface by examining the combined influence of melting heating transfer and second-order slip on a 2D-flow. Mabood and Das^14^ further make an extension of^13^ to include nonlinear thermal radiation. They were able to reveal that the Nusselt number significantly enhanced with the escalating melting parameter. Recently, temperature dependent thermal conductivity for fluid flow has been investigated in^15^ and^16^. The heat and mass transfer for second grade fluid flow and micropolar hybrid nanofluid flow over stretching sheet in magnetic field was analysed by Awan et al.^17^ and Ai-Hanava et al.^18^. Mabood et al.^19^ explored this idea to investigate the impact of thermal radiation on MHD Casson fluid flow in the presence of porous materials over a moving sheet. In a similar study, Makinde et al.^20^ addressed the behavior of magnetohydrodynamic flow and heat transfer of a Casson fluid past a stratified melting surface with variable fluid property. Very recently, Mabood et al.^21^ considered a surface with a melting heat effect on a Sisko nanofluid with nonlinear thermal radiation. Studies have shown that the impact of forced convection with thermal radiation has been of a relevant field where high temperature is required. The nonlinear thermal radiation has lately been introduced to simulate the heat transfer phenomenon which has impacted significantly on the heat transfer mechanism. From the early work of Cess^22^, he reported the boundary layer flow past a vertical plate with radiation employing the Rosseland diffusion model. It is applicable in industries and technological processes such as furnaces, rocket nozzles, engines, gas turbines, and nuclear reactors. Studies^23–26^ have continuously employed the optically thick approximation to simulate the linear radiation flux under various flow geometries. However, to reveal a detailed nonlinear radiation phenomenon, in the work of Hayat et al.^27^, it is disclosed that for the augmented radiation parameter, the Nusselt number is also reduced. Ramzan et al.^28^ explore the nonlinear radiative mechanism to investigate the MHD fluid flow in the gyrotactic microorganism with a chemical reaction. Hayat et al.^29^ and Tlili et al.^30^ examined the 3D flow of viscoelastic fluid with nanomaterials, considering the nonlinear radiation effect, 2D stagnation point nanofluid flow over a stretched sheet with nonlinear radiation is studied by Soomro et al.^31^. Kumar et al.^32^ explored the impact of nonlinear thermal radiation and a second-order slip flow of a micropolar viscous Newtonian fluid through a convective stretched surface. They reported that both the radiation and temperature ratio parameters have the tendency to influence a hike in the thermal field. A Keller box technique is employed by Laxmi and Shankar^33^ to examine the impact of nonlinear radiation on a boundary layer flow with a variable magnetic field. Some pieces of literature associated with the nonlinear radiation effect are in^34–38^.

Entropy generation is as a result of temperature differences and has been of importance in the fluid dynamic society due to its usefulness in predicting the performance of engineering processes. Bejan^39^ introduced a new path of investigation in the research field “minimization of entropy generation”. In respect to this phenomenon, Makinde and Eegunjobi^40^ examined the analysis of entropy generation on the flow of couple stress fluid with nanoparticles and nonlinear radiation. Das et al.^41^ reviewed the report of entropy generation on a transient flow viscous nanofluid over accelerated stretching surface with Newtonian heating. Ellahi et al.^42,43^ are authors who have studied the thermodynamic analysis for nanoparticles suspension in various geometries. Recent articles concerning various aspects of entropy analysis, MHD and heat flow phenomena are reported in Refs.^44–56^.

From the above-mentioned studies, it is clear that the entropy generation on MHD couple stress nanofluid flow with melting, nonlinear radiation, and first-order slip has not been studied yet. The main objective of this article is to fill this gap in the literature; therefore, we presented a numerical solution. The transformed system is tackled with RKF-45 method.

## Mathematical model

**Figure 1:**
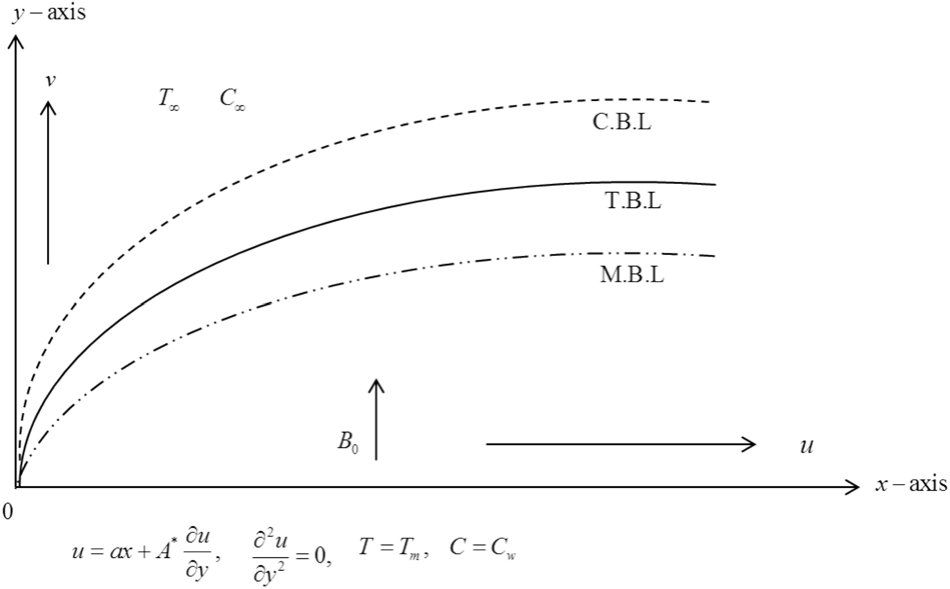
Flow geometry.

A steady slip flow, heat and mass transfer of an electrically conducted couple stressed nanofluid over a stretching sheet is considered. The analysis of heat transfer is examined through the combined influence of melting heating and thermal radiation. As shown in Fig. 1, the flow is produced by the sheet stretched with velocity $u_{w} \left( x \right) = ax$ (*a* > 0, a constant). It is further assumed that a uniform magnetic field $B_{0}$ normal to the sheet is introduced and applied parallel to *y*-axis. Here, we assumed $T_{m}$ to represent the temperature of the melting surface and $T_{\infty }$ an ambient temperature with $T_{\infty } > T_{m}$. $C_{w}$ is also taken as a nanoparticles volume fraction which is constant at stretching surface while the ambient nanoparticles volume fraction is $C_{\infty }$. The acronyms M.B.L., T.B.L. and C.B.L. represent the momentum, thermal and concentration boundary layers, respectively. The current study is essential for thermal materials processing and nano-technological fabrications. The associated equations governing are^9,14^:

1 $$ \frac{\partial u}{{\partial x}} + \frac{\partial u}{{\partial y}} = 0 $$

2 $$ u\frac{\partial u}{{\partial x}} + v\frac{\partial u}{{\partial y}} = \upsilon \frac{{\partial^{2} u}}{{\partial y^{2} }} - \frac{{\eta_{0} }}{\rho }\frac{{\partial^{4} u}}{{\partial y^{4} }} - \frac{{\sigma B_{0}^{2} u}}{\rho } $$

3 $$ \begin{aligned} u\frac{\partial T}{{\partial x}} + v\frac{\partial T}{{\partial y}} & = \frac{\partial }{\partial y}\left( {\left( {\alpha + \frac{{16\sigma_{1} }}{{3k_{1} }}T^{3} } \right)\frac{\partial T}{{\partial y}}} \right) + \frac{\mu }{{\rho C_{p} }}\left( {\frac{\partial u}{{\partial y}}} \right)^{2} + \frac{{\eta_{0} }}{{\rho C_{p} }}\left( {\frac{{\partial^{2} u}}{{\partial y^{2} }}} \right)^{2} + \frac{{\sigma B_{0}^{2} u^{2} }}{{\rho C_{p} }} \\ & \quad + \tau \left\{ {D_{m} \frac{\partial C}{{\partial y}}\frac{\partial T}{{\partial y}} + \frac{{D_{T} }}{{T_{\infty } }}\left( {\frac{\partial T}{{\partial y}}} \right)^{2} } \right\} \\ \end{aligned} $$

4 $$ u\frac{\partial C}{{\partial x}} + v\frac{\partial C}{{\partial y}} = D_{m} \frac{{\partial^{2} C}}{{\partial y^{2} }} + \frac{{D_{T} }}{{T_{\infty } }}\frac{{\partial^{2} T}}{{\partial y^{2} }} $$

The appropriate boundary conditions are:

5 $$ \begin{aligned} & u = ax + A\frac{\partial u}{{\partial y}},\quad \frac{{\partial^{2} u}}{{\partial y^{2} }} = 0\quad T = T_{m} \quad C = C_{w} \quad at\quad y = 0, \\ & u \to 0,\quad \frac{\partial u}{{\partial y}} \to 0,\quad T \to T_{\infty } ,\quad C \to C_{\infty } ,\quad {\text{as}}\quad y \to \infty \\ \end{aligned} $$

and

6 $$ k\left( {\frac{\partial T}{{\partial y}}} \right)_{y = 0} = \rho \left( {\lambda + c_{s} \left( {T_{m} - T_{0} } \right)} \right)v\left( {x,0} \right), $$

where *u* and *v* represent velocity components along the *x* and *y-*axes, respectively. $\alpha$ thermal diffusivity, $\nu$ kinematic viscosity, $\sigma$ electrical conductivity, $\rho_{f}$ density of the base fluid, $D_{T}$ thermophoresis diffusion coefficient, $D_{B}$ Brownian diffusion coefficient, $D$ diffusion coefficient, $\tau$ ratio between the nanoparticles material heat capacity and fluid heat capacity, *c* volumetric volume coefficient, $\rho_{p}$ density of the particles, and $C$ rescaled nanoparticles volume fraction, and $C_{p}$ specific heat at constant pressure.

The velocity slip is given as^32^:

7 $$ u_{slip} = \frac{2}{3}\left( {\frac{{3 - \alpha_{1} L^{3} }}{{\alpha_{1} }} - \frac{3}{2}\frac{{1 - L^{2} }}{{K_{n} }}} \right)\lambda \frac{\partial u}{{\partial y}} - \frac{1}{4}\left( {L^{4} + \frac{2}{{K_{n}^{2} }}\left( {1 - L^{2} } \right)} \right)\lambda^{2} \frac{{\partial^{2} u}}{{\partial y^{2} }} = A\frac{\partial u}{{\partial y}} + B\frac{{\partial^{2} u}}{{\partial y^{2} }} $$

where $L = \min \left( {1/K_{n} ,1} \right)$ $\alpha_{1}$ momentum accommodation coefficient with $0 \le \alpha_{1} \le 1$ and $\lambda$ molecular mean free path, $A = \frac{2}{3}\left( {\frac{{3 - \alpha_{1} L^{3} }}{{\alpha_{1} }} - \frac{3}{2}\frac{{1 - L^{2} }}{{K_{n} }}} \right)\lambda$ and $B = - \frac{1}{4}\left( {L^{4} + \frac{2}{{K_{n}^{2} }}\left( {1 - L^{2} } \right)} \right)\lambda^{2}$, where $K_{n}$ (Knudsen number $K_{n} = \lambda /L$), we have $0 \le L < 1$, therefore, *A* (> 0) and *B* (< 0) are constants.

The Rosseland approximation for radiation, we have

8 $$ q_{r} = - \frac{{4\sigma_{1} }}{{3k_{1} }}\frac{{\partial T^{4} }}{\partial y}, $$

where $\sigma_{1}$ Stefan–Boltzmann constant, and $k_{1}$ absorption coefficient $T^{4} \approx 4T_{\infty }^{3} T - 3T_{\infty }^{4}$. Combine Eqs. (3) and (8), we obtain

9 $$ \begin{aligned} u\frac{\partial T}{{\partial x}} + v\frac{\partial T}{{\partial y}} & = \alpha \frac{{\partial^{2} T}}{{\partial y^{2} }} + \frac{{16\sigma_{1} }}{{3\rho C_{p} k_{1} }}T^{3} \frac{{\partial^{2} T}}{{\partial y^{2} }} + \frac{\mu }{{\rho C_{p} }}\left( {\frac{\partial u}{{\partial y}}} \right)^{2} + \frac{{\eta_{0} }}{{\rho C_{p} }}\left( {\frac{{\partial^{2} u}}{{\partial y^{2} }}} \right)^{2} + \frac{{\sigma B_{0}^{2} u^{2} }}{{\rho C_{p} }} \\ & \quad + \tau \left\{ {D_{m} \frac{\partial C}{{\partial y}}\frac{\partial T}{{\partial y}} + \frac{{D_{T} }}{{T_{\infty } }}\left( {\frac{\partial T}{{\partial y}}} \right)^{2} } \right\} \\ \end{aligned} $$

Introducing the similarity variables^9^:

10 $$ u = axf^{\prime}\left( \eta \right),\quad \eta = \sqrt {\frac{a}{\nu }} y,\quad v = - \sqrt {av} f\left( \eta \right),\quad T = T_{m} + \left( {T_{\infty } - T_{m} } \right)\theta \left( \eta \right),\quad C = C_{\infty } + \left( {C_{w} - C_{\infty } } \right)\phi \left( \eta \right). $$

Using $T = T_{\infty } \left[ {1 + \left( {\theta_{w} - 1} \right)\theta } \right],$ where $\theta_{w} = \frac{{T_{m} }}{{T_{\infty } }}$, the governing equations take the final dimensionless form:

11 $$ f^{\prime\prime\prime} - f^{\prime 2} + ff^{\prime\prime} - Kf^{v} - Mf^{\prime} = 0 $$

12 $$ \left( {1 + \frac{4R}{3}\left( {1 + \left( {\theta_{w} - 1} \right)\theta } \right)^{3} } \right)\theta^{\prime\prime} + \Pr f\theta^{\prime} + EcPr\left( {Mf^{\prime 2} + f^{\prime \prime 2} + Kf^{\prime \prime \prime 2} } \right) + Pr\left( {N_{b} \theta^{\prime}\phi^{\prime} + N_{t} \theta^{\prime 2} } \right) = 0 $$

13 $$ \phi^{\prime\prime} + Scf\phi^{\prime} + \frac{{N_{t} }}{{N_{b} }}\theta^{\prime\prime} = 0, $$

and the transformed boundary conditions:

14 $$ f^{\prime}\left( 0 \right) = 1 + \delta f^{\prime\prime}\left( 0 \right),\quad f^{\prime\prime\prime}\left( 0 \right) = 0,\quad \theta \left( 0 \right) = 0,\quad Me\;\theta^{\prime}\left( 0 \right) + \Pr f\left( 0 \right) = 0,\quad \phi \left( 0 \right) = 1, $$

15 $$ f^{\prime}\left( \infty \right) = 0,\quad f^{\prime\prime}\left( \infty \right) = 0,\quad \theta \left( \infty \right) = 0,\quad \phi \left( \infty \right) = 0. $$

The skin friction coefficient, Nusselt, and Sherwood numbers are defined as:

16 $$ C_{f} = \frac{{\tau_{w} }}{{\rho U_{\infty }^{2} }},\quad Nu_{x} = \frac{{xq_{w} }}{{k\left( {T_{\infty } - T_{m} } \right)}},\quad Sh_{x} = \frac{{xq_{m} }}{{D_{m} \left( {C_{w} - C_{\infty } } \right)}}, $$

where

17 $$ \tau_{w} = \mu \left( {\frac{\partial u}{{\partial y}}} \right)_{y = 0} - \frac{{\eta_{0} }}{\rho }\left( {\frac{{\partial^{3} u}}{{\partial y^{3} }}} \right)_{y = 0} ;\quad q_{w} = - k\left( {1 + \frac{{16\sigma_{1} }}{{3k_{1} k}}T^{3} } \right)\left( {\frac{\partial T}{{\partial y}}} \right)_{y = 0} ;\quad q_{m} = - D_{m} \left( {\frac{\partial C}{{\partial y}}} \right)_{y = 0} . $$

Using Eq. (10), we obtain:

18 $$ \left\{ {\begin{array}{*{20}l} {\sqrt {{\text{Re}}_{x} } C_{fx} = f^{\prime\prime}\left( 0 \right) - Kf^{iv} \left( 0 \right),} \hfill \\ {\frac{{Nu_{x} }}{{\sqrt {{\text{Re}}_{x} } }} = - \left( {1 + \frac{4R}{3}\left( {1 + \left( {\theta_{w} - 1} \right)\theta \left( 0 \right)} \right)^{3} } \right)\theta^{\prime}\left( 0 \right),} \hfill \\ {\frac{{Sh_{x} }}{{\sqrt {{\text{Re}}_{x} } }} = - \phi^{\prime}\left( 0 \right).} \hfill \\ \end{array} } \right. $$

where ${\text{Re}}_{x} = \frac{{u_{w} x}}{\upsilon }$ is the local Reynolds number.

## Entropy generation

The local entropy generation analysis in dimensional form is expressed as:

19 $$ \begin{aligned} E_{G} & = \frac{k}{{T_{\infty }^{2} }}\left( {1 + \left( {\frac{{16\sigma_{1} }}{{3k_{1} k}}T^{3} } \right)} \right)\left( {\frac{\partial T}{{\partial y}}} \right)^{2} + \frac{\mu }{{T_{\infty } }}\left( {\frac{\partial u}{{\partial y}}} \right)^{2} + \frac{{\sigma B_{0}^{2} u^{2} }}{{T_{\infty } }} + \frac{{\eta_{0} }}{{T_{\infty } }}\left( {\frac{{\partial^{2} u}}{{\partial y^{2} }}} \right)^{2} + \frac{{R_{g} D_{m} }}{{C_{\infty } }}\left( {\frac{\partial C}{{\partial y}}} \right)^{2} \\ & \quad + \frac{{R_{g} D_{m} }}{{T_{\infty } }}\left( {\frac{\partial C}{{\partial y}}\frac{\partial T}{{\partial y}}} \right). \\ \end{aligned} $$

Using (10); Eq. (19) yields

20 $$ N_{G} = \underbrace {{\left( {1 + \frac{4R}{3}\left( {1 + \left( {\theta_{w} - 1} \right)\theta } \right)^{3} } \right)\theta^{\prime 2} }}_{{N_{1} }} + \underbrace {{\frac{Br}{{\Omega }}\left( {f^{\prime \prime 2} + Mf^{\prime 2} + Kf^{\prime \prime \prime 2} } \right)}}_{{N_{2} }} + \underbrace {{\Lambda \left( {\frac{{\Pi }}{{\Omega }}} \right)^{2} \phi^{\prime 2} + \Lambda \left( {\frac{{\Pi }}{{\Omega }}} \right)\theta^{\prime}\phi^{\prime}}}_{{N_{3} }} $$

The irreversibility ratio $\Phi$ is defined as:

21 $$ \Phi = \frac{{N_{2} }}{{N_{1} }} = \frac{{\frac{Br}{{\Omega }}\left( {f^{\prime \prime 2} + Mf^{\prime 2} + Kf^{\prime \prime \prime 2} } \right)}}{{\left( {1 + \frac{4R}{3}\left( {1 + \left( {\theta_{w} - 1} \right)\theta } \right)^{3} } \right)\theta^{\prime 2} }}. $$

The expression for Bejan $Be$ number for the current study is:

22 $$ Be = \frac{{N_{1} }}{{N_{G} }} = \frac{1}{1 + \Phi },\quad N_{G} = N_{1} + N_{2} + N_{3} , $$

23 $$ Be = \frac{{\left( {1 + \frac{4R}{3}\left( {1 + \left( {\theta_{w} - 1} \right)\theta } \right)^{3} } \right)\theta^{\prime 2} }}{{\left( {1 + \frac{4R}{3}\left( {1 + \left( {\theta_{w} - 1} \right)\theta } \right)^{3} } \right)\theta^{\prime 2} + \frac{Br}{{\Omega }}\left( {f^{\prime \prime 2} + Mf^{\prime 2} + Kf^{\prime \prime \prime 2} } \right) + \Lambda \left( {\frac{{\Pi }}{{\Omega }}} \right)^{2} \phi^{\prime 2} + \Lambda \left( {\frac{{\Pi }}{{\Omega }}} \right)\theta^{\prime}\phi^{\prime}}}. $$

where

$$K = \frac{{\eta_{0} a}}{{\rho \upsilon^{2} }}$$, $$M = \sqrt {\frac{{\sigma B_{0}^{2} }}{a\rho }}$$, $$R = \frac{{4\sigma_{1} T_{\infty }^{3} }}{{k_{1} k}}$$, $$Pr = \frac{{\upsilon C_{p} }}{k}$$, $$Ec = \frac{{U_{w}^{2} }}{{C_{p} \left( {T_{w} - T_{\infty } } \right)}}$$, $$N_{b} = \tau \frac{{D_{m} \left( {C_{w} - C_{\infty } } \right)}}{\upsilon }$$, $$\delta = A {\sqrt {\frac{a}{v}}}$$$$N_{t} = \tau \frac{{D_{T} \left( {T_{\infty } - T_{m} } \right)}}{{\upsilon T_{\infty } }}$$, $$Sc = \frac{\upsilon }{{D_{B} }}$$, $$Me = \frac{{c_{f} \left( {T_{\infty } - T_{m} } \right)}}{{\lambda + c_{s} \left( {T_{m} - T_{0} } \right)}}$$, $$\Lambda = \frac{{R_{g} D_{m} C_{\infty } }}{k}$$, $$\prod = \frac{{C_{w} - C_{\infty } }}{{C_{\infty } }}$$, $$\Omega = \frac{{T_{w} - T_{\infty } }}{{T_{\infty } }}$$.

## Numerical solution

Nonlinear system (11)–(13) subject to (14, 15) is computed numerically by Runge–Kutta–Fehlberg fourth fifth (RKF-45) method. The essential characteristics of operating parameters on velocity $f^{\prime}\left( \eta \right)$, temperature $\theta \left( \eta \right)$, concentration $\phi \left( \eta \right)$, skin friction coefficient, Nusselt and Sherwood numbers, Entropy generation rate $N_{G}$, and Bejan number $Be$ are represented graphically. Table 1 validates the present work with previously published results^19,57^ as a special case. The favorable agreement is observed which validate our results. The detail of the Runge–Kutta–Fehlberg fourth fifth order numerical method is given below:

24 $$ \begin{aligned} & k_{0} = f\left( {x_{i} ,\,y_{i} } \right),\quad k_{1} = f\left( {x_{i} + \frac{1}{4}h,\,y_{i} + \frac{1}{4}hk_{0} } \right),\quad k_{2} = f\left( {x_{i} + \frac{3}{8}h,\,y_{i} + \left( {\frac{3}{32}k_{0} + \frac{9}{32}k_{1} } \right)h} \right), \\ & k_{3} = f\left( {x_{i} + \frac{12}{{13}}h,\,y_{i} + \left( {\frac{1932}{{2197}}k_{0} - \frac{7200}{{2197}}k_{1} + \frac{7296}{{2197}}k_{2} } \right)h} \right), \\ & k_{4} = f\left( {x_{i} + h,\,y_{i} + \left( {\frac{439}{{216}}k_{0} - 8k_{1} + \frac{3860}{{513}}k_{2} - \frac{845}{{4104}}k_{3} } \right)h} \right), \\ & k_{5} = f\left( {x_{i} + \frac{1}{2}h,\,y_{i} + \left( { - \frac{8}{27}k_{0} + 2k_{1} - \frac{3544}{{2565}}k_{2} + \frac{1859}{{4104}}k_{3} - \frac{11}{{40}}k_{4} } \right)h} \right), \\ & y_{i + 1} = y_{i} + \left( {\frac{25}{{216}}k_{0} + \frac{1408}{{2565}}k_{2} + \frac{2197}{{4104}}k_{3} - \frac{1}{5}k_{4} } \right)h, \\ & z_{i + 1} = z_{i} + \left( {\frac{16}{{135}}k_{0} + \frac{6656}{{12825}}k_{2} + \frac{28561}{{56430}}k_{3} - \frac{9}{50}k_{4} + \frac{2}{55}k_{5} } \right)h, \\ \end{aligned} $$

where *y* is the fourth-order Runge–Kutta and *z* is the fifth-order Runge–Kutta. An estimate of the error can be obtained by subtracting the two values obtained. If the error exceeds a specific threshold, the results can be recalculated using a smaller step size. The approach to estimating the new step size is given below:

25 $$ h_{new} = h_{old} \left( {\frac{{\varepsilon h_{old} }}{{2\left| {z_{i + 1} - y_{i + 1} } \right|}}} \right)^{1/4} . $$

**Table 1:** Comparison of skin friction coefficient for various values of *M* when $Me = K = 0$, Pr = 1.

$M$	Mabood and Das^14^	Xu and Lee^57^	Present results
0	1.000008	–	1.0000000
1	− 1*.*4142135	− 1*.*41421	− 1.4142135
5	− 2*.*4494897	− 2*.*4494	− 2.4494897
10	− 3*.*3166247	− 3*.*3166	− 3.3166248
50	− 7*.*1414284	− 7*.*1414	− 7.1414284
100	− 10*.*049875	− 10*.*0498	− 10.049876
500	− 22*.*383029	− 22*.*38302	− 22.383029
1000	− 31*.*638584	–	− 31.638584

The step size and the convergence criterion are taken $10^{ - 6}$ and $\Delta \eta = 0.001$ respectively.

## Results and discussion

**Figure 2:**
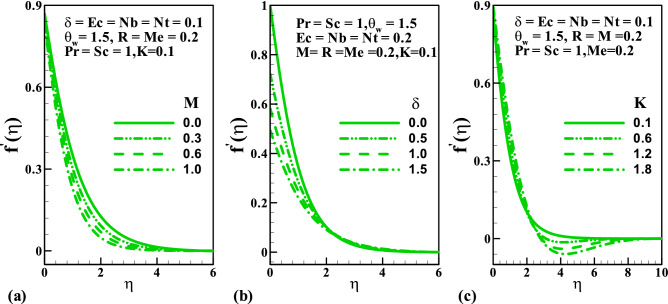
Variation of velocity with (**a**) *M*, (**b**) $\delta$, and (**c**) $K$.

**Figure 3:**
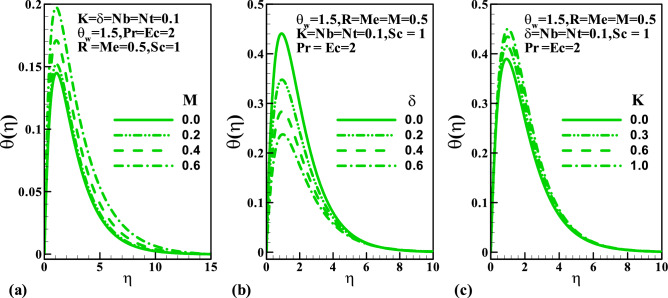
Variation of temperature with (**a**) *M*, (**b**) $\delta$, and (**c**) $K$.

We have designed “Results and discussion” to scrutinized the characteristics of the pertinent parameters like magnetic field $M$, velocity slip $\delta$, the melting $Me$, couple stress fluid $K$, radiation $R$, Eckert number $Ec$, Prandtl number $\Pr$, temperature ratio $\theta_{w}$, Schmidt number $Sc$ on the dimensionless velocity, temperature, concentration, coefficient of skin friction, Nusselt and Sherwood numbers, entropy generation due to friction and heat, total entropy generation, and the Bejan number. Figure 2 demonstrates the plots of dimensionless velocity against parameters $M$, $\delta$ and $K$. Concerning increasing the magnetic interaction, as shown in Fig. 2a, the velocity profile decelerates. This is because of an interaction of electric and magnetic fields during motion thereby producing a resistive force that acts opposite the flow direction. It is observed in Fig. 2b that the velocity significantly depreciates along the stretching wall as the slip parameter grows. This view is more pronounced at $0 \le \eta \le 2$ and maintained a uniform velocity away from the surface, while in Fig. 2c, a symmetrical nature is noticed in the velocity profile as the couple stress parameter enhances. The variation of the temperature distribution for varying values of $M$, $\delta$ and $K$ is display in Fig. 3a–c. The temperature distribution rises with an increase in the magnetic field parameter; this is perhaps due to the frictional force generated during the flow motion which is converted to heat energy. Thermal distribution lessened with a rise in the slip parameter. The lower temperature distribution is observed for viscous flow $\left( {K = 0} \right)$; therefore, due to the presence of parameter $K$ upsurge in the thermal diffusion is noticed.

**Figure 4:**
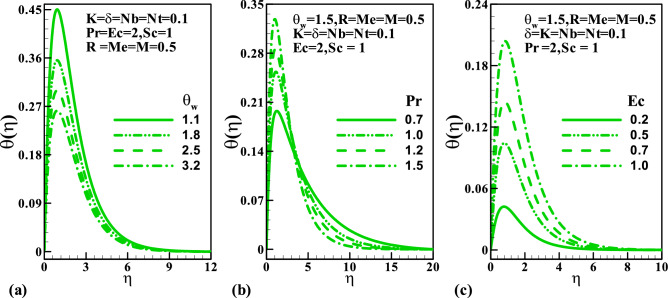
Variation of temperature with (**a**) $\theta_{w}$, (**b**) $\Pr$, and (**c**) $Ec$.

**Figure 5:**
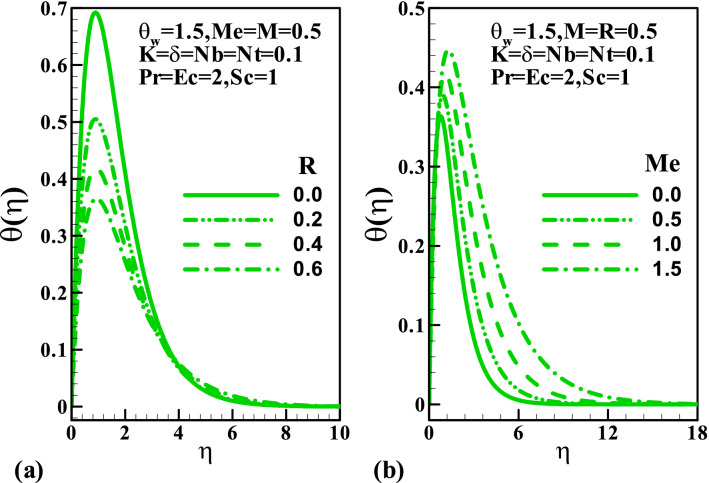
Variation of temperature with (**a**) $R$ (**b**) $Me$.

Furthermore, Fig. 4a demonstrates the behavior of temperature distribution via temperature ratio parameter $\theta_{w}$, Prandtl number $\Pr$, and Eckert number $Ec$. A rise in the parameter $\theta_{w}$ enhances the temperature distribution. Figure 4b demonstrates that the Prandtl number surges up the thermal boundary layer at the stretching wall, while a reverse trend is noticed away from the sheet. Physically, an enhancement in the Prandtl number corresponds to feeble thermal diffusivity. A significant increment in the thermal distribution is noticed for larger values of Eckert number (see Fig. 4c). Variation of radiation $R$, melting $Me$, and the Brownian motion $Nb$ parameters on the temperature are depicted in Fig. 5. From Fig. 5a, it is seen that within the range $0 \le \eta \le 3$ rise in the values of $R$ causes an increment in the temperature while a reverse trend is observed far away from the stretching wall. This is attributed to the fact that an increase in $R$ releases heat energy to the flow, thereby enhancing the temperature. The impact of the melting parameter is plotted against the temperature profile in Fig. 5b. Here, the fluid temperature elevates significantly due to a rise in the melting process. Furthermore, a gradual increment in the amplitude of the temperature profile is noticed $0 \le Nb \le 1$.

**Figure 6:**
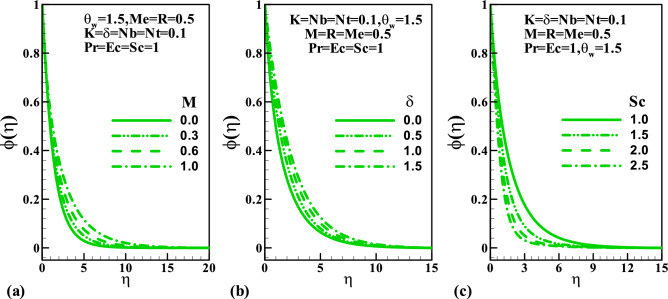
Variation of concentration with (**a**) $M$, (**b**) $\delta$, and (**c**) $Sc$.

**Figure 7:**
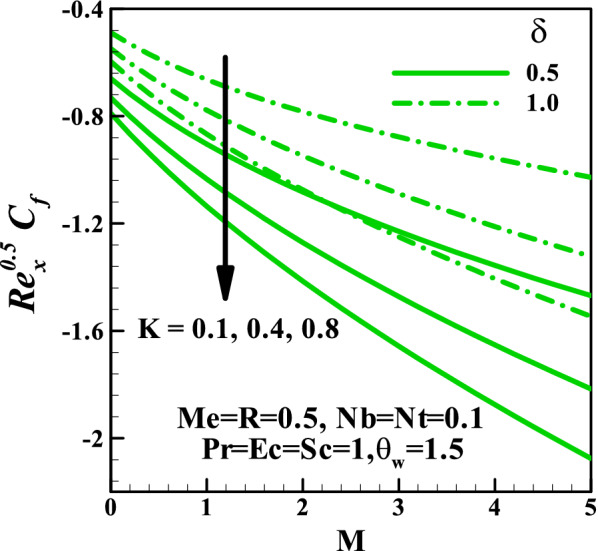
Variation of skin friction with *M* for different values of *K* and *δ*.

**Figure 8:**
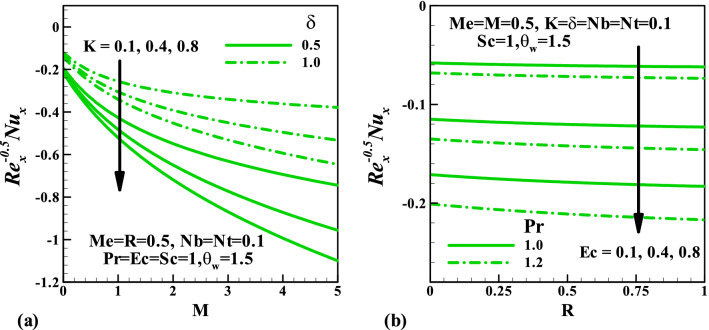
Variation of Nusselt number with (**a**) $M,\,\,K,\,\,\delta$ and (**b**) $R,\,\,Ec,\,\,\Pr$.

**Figure 9:**
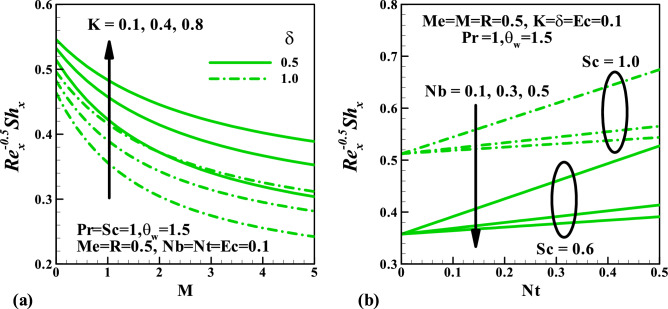
Variation of Sherwood number with (**a**) $M,\,\,K,\,\,\delta$ and (**b**) $Nt,\,\,Nb,\,\,Sc$.

Figure 6 depicts the behavior of the magnetic parameter $M$, slip parameter $\delta$, Schmidt number $Sc$ against concentration distribution. Both parameters $M$ and $\delta$ enhance the concentration distributions while the reverse trend is noticed $Sc$. This can be explained from the definition of Schmidt number $Sc = \frac{\nu }{D}$ thus, for more significant value $Sc$, the molecular diffusion rate reduces which is responsible for the reduction of concentration field. The impact of skin friction against the magnetic field parameter $M$ in response to the couple stress fluid parameter $K$ and velocity slip parameter $\delta$ is sketched in Fig. 7. This plot shows that at all values of $M$ and $K$, the skin friction suppresses, whereas it increases due to a rise in velocity gradient with $K$. Figure 8 demonstrates the features of the Nusselt number against the magnetic field parameter $M$ and radiation parameter $R$ for varying values of the couple stress fluid parameter $K$, velocity slip parameter $\delta$, Prandtl number $\Pr$ , and Eckert number $Ec$. We observed in plot Fig. 8a that increasing values of $\delta$ augments the heat transfer rate is a result of growth in the temperature gradient, whereas it diminishes with $K$. Figure 8b shows that an enhancement in both $\Pr$ and $Ec$ reduce the Nusselt number. Figure 9 graphically illustrated the behavior of the Sherwood number against the magnetic field parameter $M$ and thermophoresis parameter $Nt$ under the influences of the couple stress fluid parameter $K$, velocity slip parameter $\delta$, Brownian motion parameter $Nb$, and Schmidt number $Sc$, respectively. It is reported in Fig. 9a that an increase in $K$ enhancing the Sherwood number at each value of $M$ this may be attributed to a rise in a concentration gradient, whereas it reduces with $\delta$. The result in Fig. 9b shows that for varying values of $Nb$ the Sherwood number diminished, whereas it rises with $Sc$ regardless of $Nb$.

**Figure 10:**
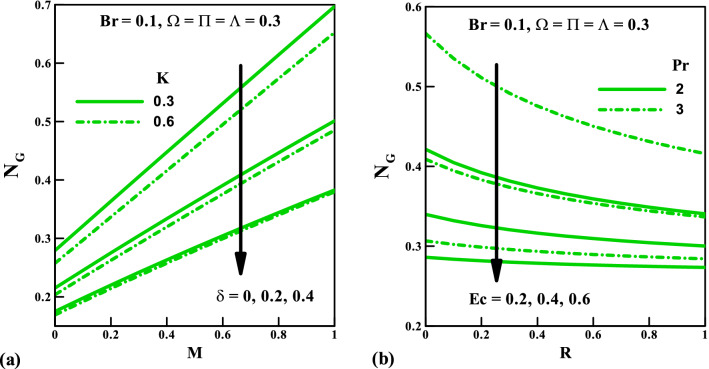
Entropy generation rate *N*_*G*_ vs. (**a**) $K,\,\,\delta$ against $M$ (**b**) $Ec,\,\,\Pr$ against $R$.

Figure 10 present the impact of the several parameters on the total entropy generation rate against the magnetic field $M$ and radiation $R$ for a varying number of couple stress fluid parameter $K$, velocity slip $\delta$, Prandtl number $\Pr$, and Eckert number $Ec$. We see in Fig. 10a that the total entropy production $N_{G}$ enhances considerably via $M$. This trend can be justified from Fig. 1 that the presence of a magnetic field generates a resistive force that helps to decelerate the flow motion. Also, across the boundary layer, the rate of entropy generation reduces for varying values of $K$ and $\delta$ (rise in $K$ and $\delta$ augment the fluid friction irreversibility). Figure 10b shows that an increased $Ec$ helps to minimize the total entropy generation, as a result of low inflow kinetic energy. Also, in the absence $\Pr$, the rate of entropy generation attains maximum and then gradually drop as $\Pr$ increases.

**Figure 11:**
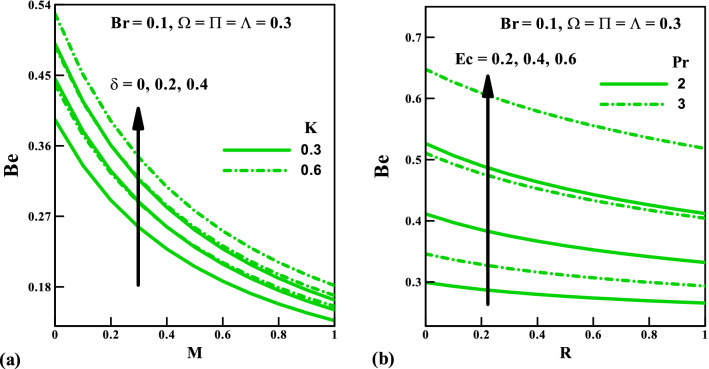
Bejan number *Be* vs. (**a**) $K,\,\,\delta$ against $M$ (**b**) $Ec,\,\,\Pr$ against $R$.

**Figure 12:**
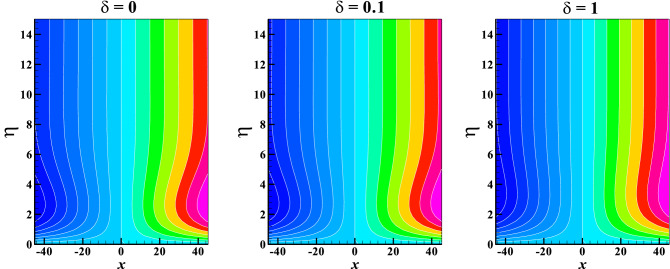
Streamlines for different values of $\delta$ when $M = Me = 0.1,\quad \theta_{w} = K = 1.2$.

**Figure 13:**
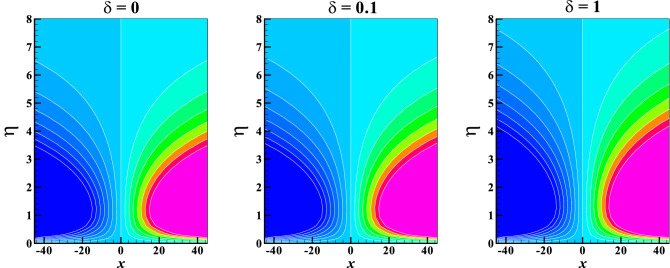
Isotherms for different values of $\delta$ when $M = Me = 0.1,\quad \theta_{w} = K = 1.2,\quad R = Ec = 0.2,\quad \Pr = 1$.

Figures 11a,b are graphically illustrated to explore the behavior of the Bejan number against the magnetic field $M$ and radiation $R$ parameters for several variables respectively. Here, a rise in the values of $M$ result to a fall in the Bejan number, this indicates a sign of a significant reduction in the irreversibility due to heat transfer. The fluid friction irreversibility upsurges for increasing values of slip parameter and couple stress fluid parameter. Furthermore, in view $\Pr$ and $Ec$ the irreversibility due to fluid friction goes up whereas the opposite effect is observed for $R$. Lastly, to show the flow pattern, the streamlines and the isotherms for various values of $\delta$ keeping other parameters fixed are presented in Figs. 12 and 13, respectively.

## Conclusions

A numerical scheme Runge–Kutta–Fehlberg fourth-fifth order is employed is to investigate for MHD couple stress fluid with nonlinear thermal radiation, velocity slip, and entropy generation numerically. The numerical features of the physical parameters are concluded as:

- Augment in $M$ leads to the diminution of flow field $f^{\prime}\left( \eta \right)$.
- Thermal field $\theta \left( \eta \right)$ and the related boundary layer get lessened with an increment in $\delta$ while the scenario is quite opposite for enhanced $Ec$.
- The Schmidt number $Sc$ is accountable in reduction of the concentration curves while increased $M$ and $\delta$ manages to enhance concentration field.
- An increase in magnetic strength and couple stress parameters augment the skin friction coefficient while opposite trend is observed for $\delta$.
- Nusselt number is increasing with $K,\,\,M$ and $Ec$.
- Sherwood number accelerates with the incremental values of $K,\,\,Nt$ and $Sc$ while an opposite scenario exists for $Nb$ and $\delta$.
- Entropy generation shows crumble for the first-order slip and Eckert number.
- Bejan number is enhanced for couple stress fluid and velocity slip parameters while opposite scenario is for radiation and magnetic parameters.

## Data Availability

The data that supports the findings of this study are available within the article.
